# Cardiovascular adverse effects of lopinavir/ritonavir and hydroxychloroquine in COVID-19 patients: Cases from a single pharmacovigilance centre

**DOI:** 10.21542/gcsp.2021.11

**Published:** 2021-06-30

**Authors:** Ioanna Istampoulouoglou, Barbara Zimmermanns, Tanja Grandinetti, Catia Marzolini, Annette Harings-Kaim, Sarah Koechlin-Lemke, Irene Scholz, Stefano Bassetti, Anne B Leuppi-Taegtmeyer

**Affiliations:** 1 Department of Clinical Pharmacology & Toxicology, University Hospital Basel, Switzerland; 2 Regional Pharmacovigilance Centre Basel, University Hospital Basel, Switzerland; 3 Division of Infectious Diseases & Hospital Hygiene, University Hospital Basel, Switzerland; 4 Swissmedic, Schweizerisches Heilmittelinstitut, Bern, Switzerland; 5 Division of Internal Medicine, University Hospital Basel, Switzerland; 6 University of Basel, Basel, Switzerland

## Abstract

In this article we summarize the cardiovascular adverse events that were observed in three patients during their treatment for COVID-19 and discuss their association with lopinavir/ ritonavir (LPV/r) and hydroxychloroquine (HCQ). The cases were reported to our regional pharmacovigilance centre in April 2020. All three patients were above 75 years in age, male and multimorbid, and had been hospitalized for treatment of COVID-19. As part of their treatment, all of them received a very strictly monitored off-label therapy with LPV/r and HCQ, for which they had given their prior, written, informed consent. In one patient, erythromycin was also administered. All three patients developed a significant QTc time prolongation during or shortly after therapy with the above drugs. On account of this, the treatment had to be discontinued early in each case and QTc time recovered in all three patients.

## Introduction

The antiretroviral drugs lopinavir/ritonavir (LPV/r) and the antimalaria drug hydroxychloroquine (HCQ) were initially proposed as potential treatments of COVID-19. Based on currently available data, the benefit-risk profile of these drugs cannot be considered favourable because they did not demonstrate efficacy and there are concerns about their safety^1^. Prolongation of the QTc interval and the associated increased risk of sudden cardiac death are the most serious adverse reactions^2,3^.

With the exception of a few recommendations^4^, no official guidelines currently exist for handling the risk of drug-induced QTc time prolongation in clinical practice, or for monitoring the QTc interval during therapy with QTc-prolonging drugs. Therefore, management is not uniform and differs among institutions and practitioners. In addition to a baseline ECG, some doctors perform an additional ECG check after the first dose when the maximum plasma concentration of the relevant drug has been reached, while others carry out a further ECG check only after the steady state of the relevant drug has been reached.

Additionally, it is known, that COVID-19 itself can affect the cardiovascular system and that viral infections predispose patients to cardiac arrhythmia due to metabolic dysfunction, myocardial inflammation and activation of the sympathetic nervous system. Consequently, the development of cardiac arrhythmia in COVID-19 patients is increased^2,3,6^.

In this article we summarize the cardiovascular adverse events that were observed in three patients during their treatment for COVID-19 and discuss their association with LPV/r and HCQ. The cases were reported to our regional pharmacovigilance centre in April 2020. There, pharmacovigilance assessments according to the World Health Organisation (WHO) criteria were performed^8^.

A spontaneous pharmacovigilance report is a snapshot that provides information on whether a drug or vaccine could in principle be associated with a suspected adverse effect. It also assesses the degree of association between the drug and the suspected adverse drug reaction (ADR), namely as certain, likely, possible, unlikely or unclassifiable. It must be noted that a spontaneous report cannot answer the question whether the symptoms observed in a particular patient are wholly attributable to the drug intake. This question can only be answered by an individual medical opinion. Nevertheless, in Switzerland, medical professionals and all those who manufacture, administer or dispense therapeutic medicinal products – including medical personnel who are authorised to do so – are obliged by law to report the occurrence of ADRs to the national regulatory authority for medicinal products, Swissmedic.

## Case presentations

The relevant demographic, clinical, and laboratory information for each patient case and details about each adverse drug reaction (ADR) as well as the outcome and ultimate pharmacovigilance assessment are given in Table 1.

**Table 1:** Demographic, clinical, and laboratory characteristics of the clinical cases and ADR details.

Patient number	Age/Sex	COVID-19 diagnosis	Comorbidities	Suspected drugs	ADR	Latency to ADR	Intervention	QTc-maximum values (latency time)	WHO causality assessment^8^	ADR outcome at time of PV report
1.	80-year-old male	Bilateral pneumonia & severe ARDS	1–2, 6	LPV/r, HCQ LPV-trough level 26.9 mg/l (day 3 of combination therapy)	QTc time prolongation from baseline value of 417 msec	Day 3 of combination therapy	LPV/r and HCQ stopped after 3 days of combination therapy	500 msec (5 days after triple combination therapy-stop)	Possible for all drugs, alone or in combination	Recovered without sequelae
2.	82-year-old male	Enteritis, bilateral pneumonia & severe ARDS	1–5	Erythromycin, LPV/r, HCQ LPV-trough level 30.1 mg/l (day 3 of LPV/r therapy)	QTc time prolongation from baseline value of 416 msec	Day 2 of erythromycin Day 9 of LPV/r 9 days after end of HCQ	Erythromycin and LPV/r stopped after 2 & 9 days of therapy, respectively	570 msec (1 day after stopping erythromycin- & LPV/r and 10 days after stopping HCQ)	Possible for all drugs, alone or in combination	Recovered without sequelae
3.	77-year-old male	Pneumonia	1, 5, 7–11	LPV/r, HCQ LPV-trough level 27.0 mg/l (day 2 of LPV/r therapy) & 20.6 mg/l (day 5 of LPV/r therapy)	QTc time prolongation from baseline value of 419 msec	Day 1 of combination therapy	LPV/r and HCQ stopped after 6 & 3 days of therapy, respectively	535 msec (day 1 of triple combination therapy)	Possible for all drugs, alone or in combination	Recovering

**Notes.**

ADR: adverse drug reaction, HCQ: hydroxychloroquine (800 mg on day 1–2 and 400 mg on day 3), LPV/r: lopinavir/ritonavir (on day 1: 2 × 800/200 mg, then 2 × 400/100 mg/day for 2, 7 or 5 days in cases 1, 2 & 3 respectively), PV: pharmacovigilance, WHO: World Health Organization. Comorbidities: 1. Arterial hypertension, 2. Type 2 diabetes mellitus, 3. Hypertriglyceridemia, 4. Obesity 5. Previous stroke, 6. Previous nephrectomy (normal creatinine value), 7. Chronic renal impairment, 8. Liver cirrhosis, 9. Cardiomyopathy with pacemaker implantation, 10. Recurrent acute polyarticular gout attacks, 11. Lumbovertebral pain syndrome.

All three patients were above 75 years in age, male and multimorbid, and had been hospitalized for treatment of COVID-19. As part of their treatment, all of them received an off-label therapy with LPV/r and HCQ, for which they had given their prior, written, informed consent. In one patient, erythromycin was also administered. All three patients developed a significant QTc time prolongation during or shortly after therapy with the above drugs. On account of this, the treatment had to be discontinued early in each case and QTc time recovered in all three patients.

The first case relates to an 80-year-old male patient hospitalized with bilateral pneumonia and severe ARDS due to a COVID-19 infection, for whom a combination therapy with LPV/r and HCQ was initiated nine days after the initial symptoms developed. Two days later, significant QTc time prolongation was observed and so LPV/r and HCQ were stopped. An intervention for the ECG changes was not necessary. Patients were continuously assessed for arrhythmias, which did not occur in any cases. The QTc time was monitored using 12-lead ECGs and returned to normal within two weeks. The patient recovered from COVID-19 and could be transferred to rehabilitation. His treatment also included tocilizumab and amoxicillin/clavulanic acid, which were not judged to be associated with the QTc time prolongation.

The second case was an 82-year-old male patient hospitalized with enteritis, bilateral pneumonia and severe ARDS due to COVID-19 infection. He developed QTc time prolongation, which was attributed by his treating doctor as drug induced. Suspected drugs were LPV/r, HCQ and erythromycin. Other causes or risk factors for QTc time prolongation such as underlying cardiac disease or hypokalemia in the context of COVID-19 associated enteritis were not reported. Therapy with LPV/r was terminated 1 day earlier than planned; and so was the therapy with erythromycin. The therapy with HCQ had already ended before the development of the ADR. As far as can be determined, no cardiac arrhythmias occurred and the QTc time returned to baseline.

The third case refers to a 77-year-old male patient hospitalized with underlying cardiomyopathy (VDD-pacemaker – i.e., ventricle paced, atrium and ventricle sensed – *in situ*), initially admitted with suspected atypical pneumonia. During the course of the admission the patient was diagnosed with COVID-19 pneumonia and non-invasive ventilation was necessary. The patient was sufficiently oxygenated with 4–6 liters oxygen/min via an oxygen mask. A prolonged QTc time was observed on the same day that therapy with LPV/r and HCQ was initiated. HCQ was continued for two more days and LPV/r for five more days. There were no reports of arrhythmia and the patient made a complete recovery, despite requiring a period of haemodialysis for acute renal failure of multifactorial origin.

## Pharmacovigilance discussion

LPV/r, HCQ, and erythromycin can all prolong the QTc interval. HCQ and erythromycin are known to cause relevant QTc time prolongation, which according to the latest AHA Recommendations for the Standardization and Interpretation of the Electrocardiogram, is a QTc time ≥ 460 msec in females and ≥ 450 msec in males^7^; QTc times > 500 msec are generally considered to carry a high risk for torsades de pointes (TdP) arrhythmia^15^. Although TdP usually ends spontaneously, it can rarely turn into ventricular fibrillation and cardiac arrest with a potentially lethal outcome.

The mechanism of QTc time prolongation by LPV/r, HCQ and erythromycin is through inhibition of the cardiac hERG (human ether-a-go-go related gene) potassium channels^13^. Inhibition of hERG channels is largely dose-/concentration-dependent and can be additively increased if hERG blockers are given together^13^. If QTc time increases by more than 60 msec or over 500 msec, the causative drugs should be discontinued right away^14^.

The following cardiac ADRs are described in the drug product information for LPV/r^10^: as occurring occasionally: atherosclerosis resulting in myocardial infarction, atrioventricular conduction disorder or tricuspid regurgitation. According to post-marketing experience, cases of bradyarrhythmia are also known, but cases of relevant QTc time prolongation, TdP or ventricular fibrillation have not been reported. Based on a controlled, randomized cross-over study in healthy volunteers^10^ receiving therapeutic doses (400/100 mg LPV/r 2x/day) or supratherapeutic doses (800/200 mg 2x/day), the maximum mean difference (upper limit 95% confidence interval) of the QTc interval with 400/100 mg LPV/r twice daily compared to placebo was 3.6 (6.3) msec; and 13.1 (15.8) msec for supratherapeutic doses of 800/200 mg LPV/r twice daily compared to placebo. QTc time prolongation of ≥ 60 msec from baseline or a QTc in excess of 500 msec did not occur. The product information for LPV/r also points out an increased risk of cardiac complications when combined with drugs known to induce a prolongation of the QTc interval, such as erythromycin or HCQ. According to Eudravigilance data, a QTc time prolongation was described in 17 spontaneous case reports during LPV/r therapy for COVID-19^3^. The global WHO database for reporting ADRs has so far received 25 reports of QTc time prolongation and 7 cases of TdP ventricular arrhythmia following LPV/r therapy out of a total 12,313 reports since 2001^16^. Overall 1,996 ADRs were reported in association with LPV/r in 2020 compared to 424 in 2019 (approximately a 5-fold increase), consistent with widespread use of this drug during the COVID-19 pandemic^16^.

ECG changes are labelled as occasional ADRs in the product information for HCQ^11^. There are warnings that HCQ may increase the QTc interval in patients with specific clinical risk factors (e.g., heart disease, cardiac arrhythmias, hypokalemia, hypomagnesemia) and when there is simultaneous treatment with substances which also prolong the QTc interval. Dose-dependency with regard to the extent to which the QTc interval is prolonged has been described. The dose in this case was the same as that which is used to treat malaria (1 × 800 mg, then 400 mg/day for 2–3 days). The global WHO database for reporting ADRs has so far received 47 reports of QTc time prolongation and 78 reports of torsades de pointes ventricular arrhythmia in association with HCQ therapy, out of a total of 29,487 reports since 1968^16^. Overall ADR-reporting increased 1.6-fold between 2019 and 2020, consistent with widespread use of this drug during the COVID-19 pandemic^16^.

In the Swiss product information for erythromycin, a QTc time prolongation is listed as a rare ADR and simultaneous use of drugs that can also lead to a prolongation of the QTc interval is listed as a contraindication^12^.

LPV is highly metabolized by the hepatic cytochrome P450 (CYP450) system, primarily by the isoenzyme CYP3A and ritonavir to a lesser extent additionally by CYP2D6. Ritonavir, a potent inhibitor of CYP3A, inhibits the metabolism of LPV, leading to an increase in plasma levels of LPV (so-called “pharmaco-enhancement”). The effective half-life of LPV in this combination is 5–6 hours (steady state is reached after 25–30 hours). The half-life of ritonavir is 3–5 hours (steady state reached after 15–25 hours) so that in theory, concentration-dependent ADRs related to LPV/r can be observed as early as during the first two days of therapy and cease within a few hours to a few days after drug discontinuation. The target LPV trough level at steady state (before the 5th administration) was > 10 mg/l for the treatment of SARS-CoV-2 with LPV/r at the centre which reported the cases. All patients reported here had LPV trough levels exceeding this target, in keeping with the observation by Marzolini and colleagues, that LPV trough levels are approximately 3.5 times higher in patients with COVID-19 compared to patients with HIV infection^5^.

LPV/r are predominantly metabolized and eliminated via the liver, so that increased plasma concentrations and increased liver toxicity are to be expected in patients with liver dysfunction. As COVID-19 can cause liver dysfunction, this might be one explanation for the reduced LPV clearance observed in these patients^9^. A more likely explanation for the reduced LPV clearance is COVID-19-associated inflammation, which inhibits drug metabolism, particularly through inhibiting the activity of CYP3A4^5^. Since excretion of LPV via the kidney is negligible, no reduced overall clearance is to be expected in patients with renal insufficiency. At present, there are no data for ritonavir for this specific group of patients.

**Figure 1.**
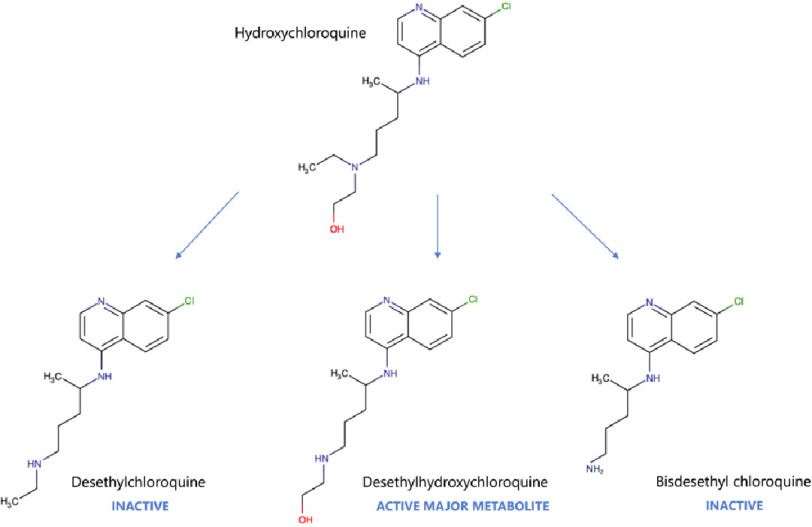
Major pathways of hydroxychloroquine metabolism.

HCQ is predominantly renally eliminated (69%) and partially metabolized by CYP2C8 and CYP3A4 (see Figure 1)^17,18^. The elimination kinetics follow a two-compartment model. For this reason, ADRs related to HCQ can in theory occur not only during therapy but also a few days to two months after stopping the drug.

Erythromycin is metabolized by the hepatic CYP450 system, primarily by the isoenzyme CYP3A4 and to a lesser extent by CYP2B6. Moreover, erythromycin is a moderate inhibitor of CYP3A4. It is mainly eliminated via the liver and gallbladder and due to its short plasma half-life of 1–2 hours, ADRs related to erythromycin can happen on the first day of therapy.

The combination of LPV/r, HCQ and erythromycin is problematic both due to pharmacokinetic and pharmacodynamic drug-drug interactions. Combining HCQ and erythromycin – two known QTc-prolonging drugs – with LPV/r, a drug with potential QTc time prolongation, can result in an additively increased risk of QTc time prolongation and TdP.

Ritonavir and erythromycin, which are substrates and inhibitors of CYP3A4 at the same time, inhibit both their metabolism and that of lopinavir, another CYP3A4 substrate, leading to a significant higher plasma level of LPV and erythromycin; and subsequently increased risk of ADR, particularly additive QTc time prolongation. The fact that COVID-19-associated inflammation itself predisposes to cardiac arrhythmia and inhibits the activity of CYP3A4, potentiates the risk of drug induced-QTc prolongation even more as evidenced by the increased reporting of QTc prolongation during the COVID-19 pandemic, particularly among male patients^19^.

## Clinical implications for prescribing and monitoring

Before prescribing QTc-prolonging drugs, not only should the potential risk of QTc time prolongation associated with the proposed drug and of all current co-medication be taken into account, but also patient-related and in part modifiable risk factors.

These risk factors include the female sex, age > 65 years, structural heart diseases, such as heart failure with low ventricular ejection fraction, left ventricular hypertrophy, and myocardial infarction, an impaired hepatic or renal function, an uncorrected electrolyte disturbance like hypokalemia, hypomagnesemia or hypocalcemia and an untreated hypothyroidism^4^.

Potassium, magnesium and calcium values, as well as the thyroid function, were not reported in our three patients. However, risk factors included older age, while one (the third) patient also had several relevant comorbidities (i.e., cardiomyopathy, pacemaker, chronic renal insufficiency and liver cirrhosis). Such patients with severe COVID-19 progression and multiple risk factors (heart disease, severe renal and liver impairment) are generally predisposed for an increased ADR rate. In addition, when several QTc-prolonging drugs are administered concurrently, the risk of QTc time prolongation increases even more due to potential drug-drug interactions. Since the QTc time peak can occur with a delay, patients must be closely monitored by ECG after administration of one and especially several QTc-prolonging drugs. In addition to determining the QTc time before the start of therapy, we also recommend further measurements, namely at the time when peak concentration (C_max_) is reached after the first administration of the implicated drug (T_max_) and after pharmacokinetic steady state is attained, so that a relevant QTc time prolongation can be detected early enough. The QTc time should ideally be monitored until the QTc time returns to pre-treatment values.

In conclusion, we report three cases of marked QTc prolongation episodes in patients with severe COVID-19 who received LPV/r and HCQ and which were registered by our pharmacovigilance centre. The analysis of individual risk factors suggest that these drugs possibly contributed to the risk of cardiovascular complications in our three patients with severe COVID-19. Since then, these repurposed drugs failed to show any benefit in the outcome of COVID-19 and so no longer have a place in treatment.
